# Dataset of TPACK in teaching practice: Adversity quotient, attitude computer technology and self-efficacy among Indonesian teachers

**DOI:** 10.1016/j.dib.2022.108749

**Published:** 2022-11-13

**Authors:** Wibowo Heru Prasetiyo, Beti Indah Sari, Noor Banu Mahadir Naidu, Halimah Sa'diyah, Rizky Novia Saputri, Jagad Aditya Dewantara, Patmisari

**Affiliations:** a Civic Education Department, Universitas Muhammadiyah Surakarta, Surakarta, Indonesia; b Social Science Education, IAIN Tulungagung, Tulungagung, Indonesia; c Moral, Civics, and Character Building Studies, Universiti Pendidikan Sultan Idris, Tanjong Malim, Malaysia; d Civic Education Department, Universitas Tanjungpura, Pontianak, Indonesia

**Keywords:** TPACK, Adversity quotient, Attitude computer technology, Self-efficacy

## Abstract

This dataset describes the measurement of adversity quotient (AQ), attitude computer technology (ACT), and self-efficacy with computer technology (SCT) of Indonesian teachers in implementing the technological pedagogical content knowledge (TPACK) concept in their teaching practice. The online survey was distributed to collect data on demographic information (4 items), AQ (11 items), ACT (19 items), SCT (22 items), and TPACK (5 items). It was carried out from August to September 2022. A total of 901 teachers from 28 provinces in Indonesia were recruited using probability sampling technique. Data from the survey were analyzed using the statistical analysis of One Way Anova and Partial Correlation. This dataset can help teacher institutions design effective programs to develop teacher digital competencies in integrating technology. Future researchers can compare this dataset with more rigorous data from developing countries.

**Specifications Table**

**Table:** 

Subject	Social Sciencies
Specific subject area	Citizenship education, Educational techology
Type of data	Table Figure
How the data were acquired	Online survey. The data were imported to Microsoft Excel and analyzed using multivariate analysis.
Data format	Raw Analyzed Descriptive
Description of data collection	901 respondents were recruited from August to September 2022. Primary data were collected from online questionnaires (Google Form).
Data source location	Data were gathered from Indonesian teachers in 28 provinces in Indonesia.
Data accessibility	Dataset is uploaded on Mendeley Repository name: Mendeley Data identification number: https://dx.doi.org/10.17632/bdzjtff5bh.2 Direct URL to data: https://data.mendeley.com/datasets/bdzjtff5bh/2

## Value of the Data

• The data were collected in the context of Indonesia where Covid-19 pandemic is changing learning practices that encourage technology integration to meet the demands of digital competence for teachers.
• The dataset is useful for teachers to develop and improve their digital competence and creativity regarding the use of technology as a form of adversity quotient and adaptation to all changes and developments in learning technology due to the impact of the Covid-19 pandemic.
• This dataset is useful for policymakers in formulating teacher education programs that help student teachers to understand the motivations and efforts to improve their competencies in developing knowledge, skills, and experiences.
• The data are helpful for researchers who want to compare the results of this study to similar research related to digital competence and the influence of the adversity quotient of teachers in seeking and finding solutions to overcome obstacles related to technology integration in learning.

## 1 Objective

The Internet transformed the entire educational system, particularly education in the twenty-first century. All teachers in Indonesia are expected to integrate their pedagogical and digital competency skills as internet penetration raises. This dataset is crucial for assessing TPACK and its affecting aspects, such as adversity quotient, attitude toward computer technology, and self-efficacy with computer technology. According to the literature, there is few research about relationships among these variables. The dataset is influential to compare it to data from similar studies conducted in other regions. In addition, the data can inform the development of action plans, pedagogies, adjustments, or interventions to best support teacher education programs.

## 2 Data Description

In Indonesia, the Covid-19 pandemic has impacted teaching and learning practice, particularly face-to-face learning shifting to online learning, both using blended approach and game-based education [1,2]. Prior studies highlight the digital class using technology-rich design as a need for transforming learning [3,4]. However, there are few published studies that investigate the readiness of teacher education programs to implement learning technology. It is a struggle to be able to enhance outcomes and actions when developing new ways for enhancing teacher competency. For that reason, this paper presents a dataset that describes factors influencing teachers in implementing technological pedagogical content knowledge (TPACK). The data are divided into three groups: (1) demographic information, including grade level of teaching, gender, and age; (2) the determinants, including adversity quotient, attitude computer technology, self-efficacy with technology; and (3) TPACK. The survey involved 901 teachers from 28 provinces in Indonesia. All question items used to measure teachers' attitude, ability and expertise in using computer technology as a form of teacher digital competence and adversity quotient variables were measured using a Likert scale with 5-point intervals (Strongly Agree to Strongly Disagree). All demographic characteristics are presented in Table 1.

**Table 1:** Respondents’ characteristics (n = 901)

Variable	Category	Frequency	Percentage (%)	Mean	Median	Std. Deviation	Variance	Range	Min	Max	Sum
Grade level of teaching	Kindergarten	120	13.3								
	Elementary school	528	58.6	1.85	1	1.123	1.262	3	1	4	
	Junior high school	104	11.5								
	Senior high school	149	16.5								1663
	N	901	100								
Gender	Male	173	19.2								
	Female	728	80.8	1.81	2	0.394	0.155	1	1	2	1629
	N	901	100								
Age of respondents (years old)	20-30	386	42.8								
	31-40	359	39.8	1.77	2	0.801	0.641		1	4	1599
	41-50	129	14.3								
	> 50	27	3.0								
	N	901	100								

Table 1 presents the characteristics of the respondents based on demographic information. The frequency distribution for each question item contained in the questionnaire is presented in Table 2. In Table 3 to 6, the relationship between respondents' demographic characteristics and variables is illustrated in a contingency table to show the interactions between respondents' demographic variables and the adversity quotient variable, attitude computer technology, self-efficacy with computer technology and TPACK.

**Table 2:** Statistics item description of adversity quotient variable, attitude computer technology, self-efficacy with technology and digital competence in the form of TPACK implementation

			Mean		95% Confidence Interval for Mean							Skewness		Kurtosis	
Variable	Code	Item	Statistic	Std. Error	Lower Bound	Upper Bound	Var	Std Dev	Min	Max	Range	Statistic	Std. Error	Statistic	Std. Error
Adversity Quotient (AQ)	Control (C1)	I can prepare technology-based learning materials and media on difficult materials	3.80	0.02	3.75	3.84	0.51	0.71	1.00	5.00	4.00	-0.26	0.08	0.06	0.16
	C2	I can control myself when I have difficulty understanding and explaining material to students	4.17	0.02	4.13	4.21	0.37	0.60	1.00	5.00	4.00	-0.28	0.08	0.60	0.16
	C3	I always learn from the problem of using ICT media in the classroom to improve teaching skills	3.92	0.03	3.87	3.97	0.57	0.75	1.00	5.00	4.00	-0.37	0.08	0.07	0.16
	Ownership (Ow1)	The learning objectives were not achieved not because I was unable to teach, but because of the lack of ICT facilities in the classroom	3.50	0.03	3.43	3.56	0.98	0.99	1.00	5.00	4.00	-0.34	0.08	-0.32	0.16
	Ow2	The reason students find it difficult to understand the material is due to my weakness in giving explanations using ICT media	3.16	0.03	3.09	3.23	1.05	1.02	1.00	5.00	4.00	0.00	0.08	-0.73	0.16
	Reach (R1)	I can create an active discussion atmosphere in the classroom even though there is a lack of learning support facilities	4.03	0.02	3.99	4.08	0.47	0.68	1.00	5.00	4.00	-0.42	0.08	0.46	0.16
	R2	I can use appropriate learning strategies even though I am not proficient in teaching using ICT media	3.75	0.02	3.70	3.80	0.55	0.74	1.00	5.00	4.00	-0.47	0.08	0.88	0.16
	R3	I can find a quick solution when teaching materials and learning media don't work properly	3.93	0.02	3.88	3.97	0.48	0.70	1.00	5.00	4.00	-0.18	0.08	-0.13	0.16
	Endurance (E1)	I can anticipate students' boredom and lack of motivation in learning	4.04	0.02	4.00	4.09	0.44	0.67	1.00	5.00	4.00	-0.30	0.08	0.22	0.16
	E2	I can create new learning resources and learning media to complement the lack of learning facilities	3.82	0.02	3.77	3.87	0.52	0.72	2.00	5.00	3.00	-0.15	0.08	-0.28	0.16
	R3	I can provide a way to package difficult and complex material to be easily understood by students	3.82	0.02	3.78	3.87	0.51	0.71	2.00	5.00	3.00	-0.06	0.08	-0.41	0.16
Attitude Computer Technology (ACT)	Anxiety (A1)	I am confident in my ability to participate in training/workshop activities that require the use of a computer	4.32	0.02	4.28	4.36	0.40	0.63	2.00	5.00	3.00	-0.49	0.08	-0.13	0.16
	A2	I feel happy every time I learn about computer technology	4.42	0.02	4.38	4.46	0.40	0.63	2.00	5.00	3.00	-0.73	0.08	0.05	0.16
	A3	I'm not the type of person who is compatible with computer technology	4.16	0.03	4.10	4.22	0.90	0.95	1.00	5.00	4.00	-1.25	0.08	1.44	0.16
	A4	There is a fear in my mind when asked to use computer technology	4.10	0.03	4.03	4.16	1.02	1.01	1.00	5.00	4.00	-1.08	0.08	0.59	0.16
	A5	Computer technology has been confusing me	4.27	0.03	4.21	4.33	0.75	0.87	1.00	5.00	4.00	-1.36	0.08	2.06	0.16
	A6	I don't feel threatened by the impact of computer technology	3.85	0.04	3.78	3.92	1.19	1.09	1.00	5.00	4.00	-1.01	0.08	0.50	0.16
	A7	I'm anxious when using a computer because I feel like I'm going to break it	4.36	0.03	4.30	4.41	0.74	0.86	1.00	5.00	4.00	-1.63	0.08	2.95	0.16
	A8	I feel comfortable with my ability to work using a computer	4.24	0.03	4.19	4.29	0.56	0.75	1.00	5.00	4.00	-1.11	0.08	2.25	0.16
	Positive Usefulness (Up1)	Communicating with other teachers via the internet can help me become a more effective teacher	4.31	0.02	4.26	4.35	0.48	0.69	2.00	5.00	3.00	-0.67	0.08	0.03	0.16
	Up2	With the use of computer technology, I can create teaching materials to improve teaching	4.41	0.02	4.37	4.45	0.38	0.62	2.00	5.00	3.00	-0.67	0.08	0.22	0.16
	Up3	If I can use software/applications on a computer, I will be a more productive teacher	4.35	0.02	4.30	4.39	0.43	0.66	1.00	5.00	4.00	-0.74	0.08	0.78	0.16
	Up4	I can use computer technology to access many sources of information needed by teachers	4.48	0.02	4.44	4.52	0.37	0.61	2.00	5.00	3.00	-0.83	0.08	0.15	0.16
	Up5	I can use computer technology to learn about classroom management techniques	4.27	0.02	4.23	4.31	0.42	0.65	2.00	5.00	3.00	-0.48	0.08	-0.02	0.16
	Negative Usefulness (Un1)	I don't use a computer every day	3.56	0.04	3.48	3.63	1.33	1.15	1.00	5.00	4.00	-0.35	0.08	-0.86	0.16
	Un2	Using a computer means I have more work to do	3.61	0.04	3.54	3.68	1.12	1.06	1.00	5.00	4.00	-0.56	0.08	-0.23	0.16
	Un3	I don't think that a computer will be of any use to me as a teacher	3.98	0.04	3.91	4.06	1.37	1.17	1.00	5.00	4.00	-1.15	0.08	0.46	0.16
	Un4	Whatever a computer can do, I can also do well in other ways	2.97	0.03	2.90	3.04	1.07	1.04	1.00	5.00	4.00	-0.06	0.08	-0.50	0.16
	Un5	I don't see how a computer can help me learn new skills	4.08	0.03	4.02	4.14	0.89	0.94	1.00	5.00	4.00	-1.26	0.08	1.65	0.16
	Un6	Knowing how to use a computer will not help in my future teaching	4.25	0.03	4.19	4.32	0.99	1.00	1.00	5.00	4.00	-1.72	0.08	2.81	0.16

Self-Efficacy with Computer Technology (SCT)	Word Processing (Wr1)	I believe I can use MS. Word for writing letters or papers	4.62	0.02	4.58	4.66	0.35	0.59	1.00	5.00	4.00	-1.56	0.08	2.87	0.16
	Wr2	I believe I can access the saved files directly from the MS. Word	4.65	0.02	4.61	4.68	0.33	0.58	1.00	5.00	4.00	-1.72	0.08	3.71	0.16
	Wr3	I believe I can improve my writing when using MS. Word	4.64	0.02	4.60	4.68	0.33	0.58	1.00	5.00	4.00	-1.61	0.08	3.03	0.16
	Wr4	I believe I can format text (eg make text bold, underline, write italic) in MS. Word	4.70	0.02	4.66	4.73	0.29	0.54	1.00	5.00	4.00	-1.90	0.08	4.57	0.16
	Wr5	I believe I can move blocks of text while typing in MS. Word	4.61	0.02	4.57	4.65	0.39	0.62	1.00	5.00	4.00	-1.68	0.08	3.37	0.16
	Wr6	I believe I can use the spelling checker or word spell checker in MS. Word	4.19	0.03	4.14	4.25	0.66	0.81	1.00	5.00	4.00	-0.67	0.08	-0.23	0.16
	Wr7	I believe I can use the word search in MS. Word	4.45	0.02	4.40	4.50	0.52	0.72	1.00	5.00	4.00	-1.23	0.08	1.26	0.16
	Wr8	I believe I can print the file I wrote in MS. Word	4.69	0.02	4.65	4.73	0.32	0.57	1.00	5.00	4.00	-2.00	0.08	4.77	0.16
	Wr9	I believe I can save the document I wrote in MS. Word	4.73	0.02	4.70	4.77	0.26	0.51	2.00	5.00	3.00	-1.97	0.08	4.18	0.16
	Wr10	I believe renaming files in MS. Word to create backup files	4.70	0.02	4.67	4.74	0.31	0.56	1.00	5.00	4.00	-2.05	0.08	4.98	0.16
	Email (Em1)	I believe I'm able to log in to my email	4.71	0.02	4.67	4.74	0.28	0.53	2.00	5.00	3.00	-1.75	0.08	2.67	0.16
	Em2	I believe I can open the message in the email inbox	4.68	0.02	4.64	4.72	0.34	0.58	2.00	5.00	3.00	-1.91	0.08	3.65	0.16
	Em3	I believe I can reply to messages that come into my inbox	4.66	0.02	4.62	4.70	0.37	0.61	2.00	5.00	3.00	-1.82	0.08	3.02	0.16
	Em4	I believe I can delete messages received in email	4.65	0.02	4.61	4.69	0.36	0.60	2.00	5.00	3.00	-1.72	0.08	2.66	0.16
	Em5	I believe I can send the same email message to more than one person	4.50	0.02	4.45	4.54	0.51	0.71	2.00	5.00	3.00	-1.26	0.08	0.86	0.16
	Em6	I believe I can personally reply to emails from incoming emails sent to multiple people	4.43	0.02	4.39	4.48	0.56	0.75	2.00	5.00	3.00	-1.14	0.08	0.54	0.16
	Em7	I believe sure I can forward emails that come into inbox to other people	4.42	0.03	4.37	4.47	0.60	0.77	1.00	5.00	4.00	-1.22	0.08	0.89	0.16
	Em8	I believe I can log out of my email	4.65	0.02	4.61	4.69	0.38	0.61	1.00	5.00	4.00	-1.88	0.08	3.85	0.16
	CD-Rom Database (Cr1)	I believe I can use data storage hardware such as CD-ROM, floppy disk, flash disk, or hard disk	4.56	0.02	4.52	4.60	0.44	0.67	2.00	5.00	3.00	-1.54	0.08	2.25	0.16
	Cr2	I believe I can choose the right type of data storage according to the type of file	4.57	0.02	4.52	4.61	0.41	0.64	2.00	5.00	3.00	-1.39	0.08	1.62	0.16
	Cr3	I believe I can choose the right keywords when browsing data	4.52	0.02	4.47	4.56	0.44	0.66	1.00	5.00	4.00	-1.24	0.08	1.31	0.16
	Cr4	I believe I can access the files stored in the data storage hardware (CD-ROM, floppy disk, flash disk, or hard disk) and find the files I need	4.58	0.02	4.54	4.62	0.40	0.63	2.00	5.00	3.00	-1.42	0.08	1.64	0.16

TPACK	TPACK1	I can teach learning that appropriately combines subject matter, technology, and teaching approach.	4.05	0.02	4.00	4.09	0.49	0.70	2.00	5.00	3.00	-0.22	0.08	-0.45	0.16
	TPACK2	I can choose technologies for classroom learning that improve teaching content, learning processes, and student understanding.	4.10	0.02	4.06	4.15	0.45	0.67	2.00	5.00	3.00	-0.30	0.08	-0.18	0.16
	TPACK3	I can use teaching strategies that combine the right material content, technology, and teaching approaches that I learn from learning reflection assignments in class.	4.00	0.02	3.95	4.05	0.51	0.71	2.00	5.00	3.00	-0.27	0.08	-0.26	0.16
	TPACK4	I can help other teachers to combine material content, technology, and teaching approach in our school.	3.84	0.03	3.78	3.89	0.62	0.79	1.00	5.00	4.00	-0.16	0.08	-0.40	0.16
	TPACK5	I can have technology that can improve the quality of material content for a learning topic.	4.05	0.02	4.00	4.10	0.53	0.73	2.00	5.00	3.00	-0.31	0.08	-0.41	0.16

**Table 3:** Correlation between respondents' demographic characteristics and adversity quotient

		Adversity Quotient										
Demographic Variable		C1	C2	C3	Ow1	Ow2	R1	R2	R3	E1	E2	E3
Grade level of teaching	Kindergarten	3.55	4.03	3.85	3.70	2.73	3.92	3.75	3.78	3.98	3.85	3.74
	Elementary school	3.83	4.16	3.91	3.49	3.19	4.05	3.74	3.92	4.08	3.83	3.83
	Junior high school	3.76	4.21	3.90	3.42	3.25	3.98	3.62	3.95	3.98	3.69	3.71
	Senior high school	3.91	4.29	4.02	3.42	3.33	4.10	3.87	4.03	4.03	3.84	3.93
	$\bar{X}$	3.76	4.17	3.92	3.51	3.13	4.01	3.74	3.92	4.02	3.80	3.80
	F Test	6.52	4.49	1.26	2.21	8.98	2.01	2.41	2.94	1.08	1.26	2.46
	Significance	0.00	0.00	0.29	0.09	0.00	0.11	0.07	0.03	0.36	0.29	0.06

Gender	Male	3.98	4.26	4.05	3.46	3.30	4.16	3.71	4.06	4.14	3.90	3.97
	Female	3.75	4.15	3.89	3.51	3.13	4.00	3.76	3.89	4.02	3.80	3.79
	$\bar{X}$	3.87	4.20	3.97	3.48	3.21	4.08	3.73	3.98	4.08	3.85	3.88
	F Test	14.97	4.93	6.51	0.40	4.00	7.51	0.72	8.36	4.87	2.37	8.67
	Significance	0.00	0.03	0.01	0.53	0.05	0.01	0.40	0.00	0.03	0.12	0.00

Age of respondents (years old)	20-30	3.79	4.16	3.88	3.45	3.32	4.03	3.66	3.94	4.03	3.80	3.79
	31-40	3.87	4.18	3.94	3.47	3.14	4.04	3.76	3.96	4.06	3.83	3.85
	41-50	3.66	4.13	4.04	3.72	2.80	4.02	3.95	3.82	4.05	3.83	3.85
	> 50	3.56	4.26	3.78	3.59	2.89	4.15	3.89	3.81	4.07	3.96	3.85
	$\bar{X}$	3.72	4.18	3.91	3.56	3.04	4.06	3.81	3.88	4.05	3.86	3.83
	F Test	4.04	0.46	1.92	2.69	9.49	0.30	5.19	1.58	0.18	0.51	0.54
	Significance	0.01	0.71	0.13	0.05	0.00	0.83	0.00	0.19	0.91	0.68	0.66

**Table 4:** Correlation between respondents' demographic characteristics and computer technology attitude

Demographic Variable		Attitude Computer Technology																		
		A1	A2	A3	A4	A5	A6	A7	A8	Up1	Up2	Up3	Up4	Up5	Un1	Un2	Un3	Un4	Un5	Un6
Grade level of teaching	Kindergarten	4.00	4.30	3.81	3.55	3.83	3.66	4.15	3.90	4.30	4.31	4.38	4.29	4.09	3.13	3.30	3.68	2.89	3.95	4.20
	Elementary school	4.37	4.44	4.20	4.21	4.35	3.88	4.40	4.30	4.29	4.41	4.32	4.54	4.32	3.64	3.65	4.02	2.95	4.07	4.25
	Junior high school	4.34	4.42	4.14	3.99	4.18	3.97	4.30	4.21	4.28	4.39	4.31	4.42	4.24	3.40	3.70	3.85	3.05	4.05	4.26
	Senior high school	4.40	4.44	4.30	4.19	4.41	3.81	4.42	4.32	4.40	4.48	4.44	4.49	4.28	3.70	3.68	4.21	3.07	4.28	4.32
	$\bar{X}$	4.27	4.40	4.11	3.99	4.19	3.83	4.32	4.18	4.32	4.40	4.36	4.44	4.23	3.47	3.58	3.94	2.99	4.08	4.26
	F Test	12.43	1.76	7.04	15.63	14.35	1.91	3.12	10.34	1.03	1.69	1.69	5.73	4.04	7.91	4.14	5.14	1.01	2.99	0.32
	Significance	0.00	0.15	0.00	0.00	0.00	0.13	0.03	0.00	0.38	0.17	0.17	0.00	0.01	0.00	0.01	0.00	0.39	0.03	0.81

Gender	Male	4.47	4.57	4.29	4.41	4.49	4.04	4.53	4.47	4.50	4.51	4.47	4.59	4.40	3.88	3.68	3.94	2.91	4.20	4.35
	Female	4.28	4.39	4.13	4.02	4.22	3.81	4.32	4.19	4.26	4.38	4.31	4.46	4.24	3.48	3.59	3.99	2.99	4.06	4.23
	$\bar{X}$	4.38	4.48	4.21	4.22	4.35	3.92	4.42	4.33	4.38	4.45	4.39	4.52	4.32	3.68	3.64	3.97	2.95	4.13	4.29
	F Test	12.92	12.36	4.35	21.35	14.11	6.41	8.37	21.13	16.70	5.96	8.27	6.64	9.21	17.51	0.95	0.34	0.69	3.10	2.14
	Significance	0.00	0.00	0.04	0.00	0.00	0.01	0.00	0.00	0.00	0.02	0.00	0.01	0.00	0.00	0.33	0.56	0.41	0.08	0.14

Age of respondents (years old)	20-30	4.43	4.47	4.34	4.30	4.45	3.87	4.50	4.33	4.32	4.44	4.35	4.57	4.29	3.64	3.64	4.19	3.01	4.24	4.32
	31-40	4.33	4.45	4.19	4.17	4.32	3.91	4.43	4.30	4.32	4.40	4.34	4.51	4.31	3.67	3.68	3.94	2.95	4.06	4.29
	41-50	4.05	4.29	3.70	3.46	3.78	3.70	3.91	3.92	4.29	4.36	4.34	4.24	4.16	3.12	3.33	3.62	2.95	3.79	4.03
	> 50	3.89	4.04	3.30	3.26	3.44	3.56	3.48	3.70	4.00	4.19	4.30	4.04	3.96	2.96	3.56	3.30	2.85	3.59	3.85
	$\bar{X}$	4.17	4.31	3.88	3.80	4.00	3.76	4.08	4.06	4.23	4.35	4.33	4.34	4.18	3.35	3.55	3.76	2.94	3.92	4.12
	F Test	16.85	6.10	24.29	32.17	30.92	1.94	27.56	15.71	1.89	1.84	0.09	15.23	3.90	10.55	3.68	12.02	0.38	10.48	4.40
	Significance	0.00	0.00	0.00	0.00	0.00	0.12	0.00	0.00	0.13	0.14	0.97	0.00	0.01	0.00	0.01	0.00	0.77	0.00	0.00

**Table 5:** Correlation between respondents' demographic characteristics and self-efficacy with computer technology

		Self-Efficacy with Computer Technology																					
Demographic Variable		Wr1	Wr2	Wr3	Wr4	Wr5	Wr6	Wr7	Wr8	Wr9	Wr10	Em1	Em2	Em3	Em4	Em5	Em6	Em7	Em8	Cr1	Cr2	Cr3	Cr4
Grade level of teaching	Kindergarten	4.23	4.32	4.35	4.36	4.19	3.75	3.95	4.33	4.49	4.39	4.39	4.35	4.28	4.22	4.03	3.99	3.94	4.21	4.07	4.11	4.10	4.19
	Elementary school	4.68	4.70	4.69	4.76	4.69	4.23	4.51	4.75	4.77	4.77	4.76	4.74	4.72	4.72	4.55	4.49	4.50	4.71	4.65	4.64	4.59	4.65
	Junior high school	4.63	4.61	4.61	4.65	4.59	4.22	4.50	4.67	4.70	4.69	4.68	4.67	4.65	4.63	4.45	4.39	4.32	4.67	4.47	4.49	4.42	4.49
	Senior high school	4.72	4.76	4.72	4.78	4.67	4.40	4.61	4.79	4.81	4.74	4.79	4.76	4.75	4.77	4.69	4.61	4.60	4.80	4.70	4.72	4.67	4.70
	$\bar{X}$	4.57	4.59	4.59	4.64	4.53	4.15	4.39	4.63	4.69	4.65	4.66	4.63	4.60	4.58	4.43	4.37	4.34	4.60	4.47	4.49	4.44	4.51
	F Test	22.85	17.46	13.04	20.57	22.89	16.42	24.81	21.06	11.42	15.68	18.28	16.32	19.60	27.57	23.48	18.94	21.99	27.42	30.73	28.76	22.74	21.13
	Significance	0.00	0.00	0.00	0.00	0.00	0.00	0.00	0.00	0.00	0.00	0.00	0.00	0.00	0.00	0.00	0.00	0.00	0.00	0.00	0.00	0.00	0.00

Gender	Male	4.76	4.79	4.74	4.83	4.76	4.43	4.69	4.82	4.83	4.81	4.82	4.82	4.81	4.79	4.72	4.68	4.67	4.81	4.76	4.78	4.68	4.76
	Female	4.59	4.61	4.62	4.67	4.57	4.13	4.40	4.66	4.71	4.68	4.68	4.65	4.63	4.62	4.44	4.38	4.36	4.61	4.51	4.52	4.48	4.54
	$\bar{X}$	4.67	4.70	4.68	4.75	4.67	4.28	4.54	4.74	4.77	4.74	4.75	4.74	4.72	4.70	4.58	4.53	4.52	4.71	4.64	4.65	4.58	4.65
	F Test	11.69	13.68	6.41	12.40	13.35	19.38	23.37	11.60	7.26	7.73	8.51	12.22	12.85	10.71	22.34	22.94	22.63	14.37	20.51	24.73	12.75	16.95
	Significance	0.00	0.00	0.01	0.00	0.00	0.00	0.00	0.00	0.01	0.01	0.00	0.00	0.00	0.00	0.00	0.00	0.00	0.00	0.00	0.00	0.00	0.00

Age of respondents (years old)	20-30	4.74	4.75	4.76	4.81	4.75	4.29	4.62	4.82	4.83	4.83	4.85	4.84	4.83	4.80	4.67	4.62	4.62	4.81	4.72	4.71	4.67	4.73
	31-40	4.68	4.70	4.67	4.76	4.66	4.28	4.51	4.75	4.77	4.75	4.75	4.73	4.72	4.70	4.55	4.48	4.48	4.70	4.64	4.64	4.56	4.64
	41-50	4.24	4.28	4.32	4.31	4.16	3.77	3.91	4.25	4.44	4.34	4.33	4.25	4.17	4.22	3.96	3.92	3.83	4.20	4.03	4.09	4.07	4.13
	> 50	4.04	4.15	4.11	4.04	4.04	3.63	3.93	4.11	4.11	4.07	3.93	3.85	3.81	3.96	3.78	3.67	3.67	3.81	3.70	3.78	3.85	3.93
	$\bar{X}$	4.42	4.47	4.46	4.48	4.40	3.99	4.24	4.48	4.54	4.50	4.47	4.42	4.38	4.42	4.24	4.17	4.15	4.38	4.27	4.31	4.29	4.35
	F Test	36.67	32.85	29.27	49.83	42.89	20.73	41.52	50.65	36.84	41.88	60.22	63.37	69.50	50.48	48.65	43.00	49.45	59.05	61.58	53.36	41.39	44.39
	Significance	0.00	0.00	0.00	0.00	0.00	0.00	0.00	0.00	0.00	0.00	0.00	0.00	0.00	0.00	0.00	0.00	0.00	0.00	0.00	0.00	0.00	0.00

**Table 6:** Correlation between respondent characteristics and TPACK

		TPACK				
Demographic	Variable	TPACK1	TPACK2	TPACK3	TPACK4	TPACK5
Grade level of teaching	Kindergarten	3.79	3.88	3.78	3.58	3.77
	Elementary school	4.10	4.15	4.05	3.90	4.10
	Junior high school	3.97	4.02	3.92	3.74	4.03
	Senior high school	4.10	4.16	4.07	3.87	4.13
	$\bar{X}$	3.99	4.05	3.95	3.77	4.01
	F Test	7.40	6.17	5.61	6.44	7.70
	Significance	0.00	0.00	0.00	0.00	0.00

Gender	Male	4.20	4.28	4.16	4.12	4.25
	Female	4.01	4.06	3.96	3.77	4.01
	$\bar{X}$	4.11	4.17	4.06	3.94	4.13
	F Test	10.78	15.73	10.48	27.98	15.92
	Significance	0.00	0.00	0.00	0.00	0.00

Age of respondents (years old)	20-30	4.08	4.14	4.02	3.90	4.12
	31-40	4.09	4.13	4.03	3.89	4.07
	41-50	3.91	3.99	3.89	3.57	3.85
	> 50	3.67	3.81	3.81	3.48	3.81
	$\bar{X}$	3.94	4.02	3.94	3.71	3.96
	F Test	5.20	3.30	1.89	8.19	5.39
	Significance	0.00	0.02	0.13	0.00	0.00

## 3 Experimental Design, Materials and Methods

The research sample comprised teachers from 28 provinces in Indonesia and their various levels of teaching were selected by probability sampling. The sample is based on a convenient random sample for research [5]. Data collection was designed online using Google Forms due to the pandemic of Covid-19. Questionnaire links were distributed personally according to the respondents' selection criteria: having more than five years of teaching experience and having completed or enrolled in a teacher professional development program at several teacher institutions. The survey received a total of 901 responses, and all met the criteria for the statistical analysis stage.

The questionnaire was developed based on influential studies. Item questions for a variable of adversity quotient were guided by a notion from Stoltz [6]. The variables of attitude computer technology and self-efficacy with computers were developed from Milbrath and Kenzie [7]. The variable of TPACK was adapted from Schmidt et al. [8]. In the aspect of teacher demographic characteristics, descriptive statistical tests were carried out with analytical techniques using one-way ANOVA and partial correlations were carried out to determine the relationship and influence of these factors on the adversity quotient variable, computer technology attitude, self-efficacy with computer technology, and TPACK. Next, the instrument's reliability and validity analysis was carried out using statistical analysis of One Way Anova and Partial Correlation using Microsoft excel and SPSS version 25.0 application.

In the aspect of the demographic characteristics of the respondents, measurements were conducted regarding the aspects used as parameters of digital competence as well as the role and influence of the adversity quotient of teachers in integrating technology in the learning process. In this case, individual demographic characteristics are a potential source of data related to teachers' knowledge, attitudes, skills, and practices in utilizing digital technology in the learning process. The data were analyzed using frequency and percentage. The correlation analysis and One Way Anova test presented in Tables 3 to 6 describe the relationship and differences in experience and expertise as a form of teacher digital competence, including using computers to increase the effectiveness of teaching and learning. Table 7 presents data on the distribution of the frequency. In The table shows the description of the level of respondent categories in each of the variables tested. As Table 8 indicates, for all the variables measured, the majority of the distribution of responses from respondents is in the high category for the Self-efficacy with Computer Technology variable by 57%, and TPACK by 46% included in the high category. The percentage of the frequency distribution of respondents' responses is 73% for the Advertising Quotient variable and 81% for the Attitude Computer Technology variable which belong to the medium category. In conclusion, when respondents have a high level of Self-efficacy with Computer Technology and the application of TPACK in the learning process in the classroom, effective learning can be created so that it is easier for students to understand the material and achieve learning objectives.

**Table 7:** Distribution of the frequency of respondents' responses to each item

	Strongly disagree		Disagree		Neutral		Agree		Strongly agree		Total		
Item	*F*	*%*	*F*	*%*	*F*	*%*	*F*	*%*	*F*	*%*	*N*	*%*	Average
C1	1	0.11	27	3.00	252	27.97	496	55.05	125	13.87	901	100	3.80
C2	1	0.11	2	0.22	89	9.88	561	62.26	248	27.52	901	100	4.17
C3	2	0.22	24	2.66	209	23.20	474	52.61	192	21.31	901	100	3.92
Ow1	26	2.89	111	12.32	292	32.41	331	36.74	141	15.65	901	100	3.50
Ow2	33	3.66	227	25.19	288	31.96	268	29.74	85	9.43	901	100	3.16
R1	1	0.11	14	1.55	147	16.32	530	58.82	209	23.20	901	100	4.03
R2	8	0.89	23	2.55	271	30.08	485	53.83	114	12.65	901	100	3.75
R3	1	0.11	10	1.11	217	24.08	499	55.38	174	19.31	901	100	3.93
E1	1	0.11	7	0.78	153	16.98	530	58.82	210	23.31	901	100	4.04
E2	0	0.00	24	2.66	257	28.52	477	52.94	143	15.87	901	100	3.82
E3	0	0.00	18	2.00	269	29.86	469	52.05	145	16.09	901	100	3.82
A1	0	0.00	4	0.44	70	7.77	461	51.17	366	40.62	901	100	4.32
A2	0	0.00	4	0.44	57	6.33	395	43.84	445	49.39	901	100	4.42
A3	20	2.22	39	4.33	108	11.99	344	38.18	390	43.29	901	100	4.16
A4	18	2.00	65	7.21	116	12.87	316	35.07	386	42.84	901	100	4.10
A5	13	1.44	26	2.89	92	10.21	343	38.07	427	47.39	901	100	4.27
A6	48	5.33	60	6.66	143	15.87	376	41.73	274	30.41	901	100	3.85
A7	14	1.55	28	3.11	63	6.99	314	34.85	482	53.50	901	100	4.36
A8	8	0.89	11	1.22	90	9.99	439	48.72	353	39.18	901	100	4.24
Up1	0	0.00	9	1.00	91	10.10	416	46.17	385	42.73	901	100	4.31
Up2	0	0.00	5	0.55	47	5.22	426	47.28	423	46.95	901	100	4.41
Up3	2	0.22	2	0.22	75	8.32	426	47.28	396	43.95	901	100	4.35
Up4	0	0.00	3	0.33	45	4.99	367	40.73	486	53.94	901	100	4.48
Up5	0	0.00	6	0.67	82	9.10	475	52.72	338	37.51	901	100	4.27
Un1	35	3.88	152	16.87	222	24.64	260	28.86	232	25.75	901	100	3.56
Un2	37	4.11	98	10.88	231	25.64	347	38.51	188	20.87	901	100	3.61
Un3	54	5.99	68	7.55	96	10.65	304	33.74	379	42.06	901	100	3.98
Un4	77	8.55	206	22.86	341	37.85	219	24.31	58	6.44	901	100	2.97
Un5	23	2.55	45	4.99	92	10.21	415	46.06	326	36.18	901	100	4.08
Un6	36	4.00	32	3.55	49	5.44	335	37.18	449	49.83	901	100	4.25
Wr1	1	0.11	4	0.44	32	3.55	262	29.08	602	66.81	901	100	4.62
Wr2	1	0.11	5	0.55	26	2.89	247	27.41	622	69.03	901	100	4.65
Wr3	1	0.11	3	0.33	31	3.44	249	27.64	617	68.48	901	100	4.64
Wr4	1	0.11	3	0.33	22	2.44	216	23.97	659	73.14	901	100	4.70
Wr5	2	0.22	4	0.44	44	4.88	245	27.19	606	67.26	901	100	4.61
Wr6	2	0.22	16	1.78	166	18.42	340	37.74	377	41.84	901	100	4.19
Wr7	2	0.22	9	1.00	85	9.43	289	32.08	516	57.27	901	100	4.45
Wr8	1	0.11	5	0.55	28	3.11	205	22.75	662	73.47	901	100	4.69
Wr9	0	0.00	4	0.44	18	2.00	193	21.42	686	76.14	901	100	4.73
Wr10	1	0.11	4	0.44	28	3.11	195	21.64	673	74.69	901	100	4.70
Em1	0	0.00	2	0.22	29	3.22	198	21.98	672	74.58	901	100	4.71
Em2	0	0.00	7	0.78	34	3.77	197	21.86	663	73.58	901	100	4.68
Em3	0	0.00	7	0.78	44	4.88	196	21.75	654	72.59	901	100	4.66
Em4	0	0.00	6	0.67	42	4.66	211	23.42	642	71.25	901	100	4.65
Em5	0	0.00	11	1.22	83	9.21	256	28.41	551	61.15	901	100	4.50
Em6	0	0.00	15	1.66	97	10.77	271	30.08	518	57.49	901	100	4.43
Em7	1	0.11	19	2.11	97	10.77	266	29.52	518	57.49	901	100	4.42
Em8	1	0.11	7	0.78	40	4.44	209	23.20	644	71.48	901	100	4.65
Cr1	0	0.00	14	1.55	46	5.11	263	29.19	578	64.15	901	100	4.56
Cr2	0	0.00	8	0.89	49	5.44	269	29.86	575	63.82	901	100	4.57
Cr3	1	0.11	5	0.55	63	6.99	291	32.30	541	60.04	901	100	4.52
Cr4	0	0.00	7	0.78	50	5.55	257	28.52	587	65.15	901	100	4.58
TPACK1	0	0.00	8	0.89	175	19.42	485	53.83	233	25.86	901	100	4.05
TPACK2	0	0.00	8	0.89	139	15.43	507	56.27	247	27.41	901	100	4.10
TPACK3	0	0.00	15	1.66	185	20.53	487	54.05	214	23.75	901	100	4.00
TPACK4	2	0.22	25	2.77	277	30.74	412	45.73	185	20.53	901	100	3.84
TPACK5	0	0.00	13	1.44	176	19.53	463	51.39	249	27.64	901	100	4.05

**Table 8:** Frequency distribution of respondents' responses related to adversity quotient, attitude computer technology, self-efficacy with computer technology, and TPACK

Adversity Quotient				Self-Efficacy with Computer Technology			
AQ	Interval	F	%	SCT	Interval	F	%
Very high	57-68	0	0	Very high	110-132	283	31
High	45-56	224	25	High	87-109	514	57
Middle	33-44	657	73	Middle	64-86	99	11
Low	21-32	20	2	Low	41-63	5	1
Very low	9-20	0	0	Very low	18-40	0	0
Total		901	100	Total		901	100

Attitude Computer Technology				TPACK			
ACT	Interval	F	%	TPACK	Interval	F	%

Very high	95-114	4	0	Very high	25-29	134	15
High	75-94	15	2	High	20-24	418	46
Middle	55-74	733	81	Middle	15-19	325	36
Low	35-54	149	17	Low	10-14	23	3
Very low	15-34	0	0	Very low	4-9	1	0
Total		901	100	Total		901	100

## Ethics Statements

The authors ensured that the respondents provided their information voluntarily. All of the respondent's personal information is utilized for research purposes and will be kept in accordance with the anonymity principle. Prior the data collection, Universitas Muhammadiyah Surakarta has responsibility for this project by Research Ethic Committee and Granted the approval code of 215/A.3-III/FKIP/III/2022. Respondents could leave any time and for any reason, with no penalty and loss of benefits to which they were entitled, if any. The online survey was written anonymously to guarantee the confidentiality of their personal data.

## CRediT Author Statement

**Wibowo Heru Prasetiyo:** Conceptualization, Methodology, Investigation, Writing – review & editing; **Beti Indah Sari:** Concepualization, Formal analysis, Validation, Writing – original draft; **Noor Banu Mahadir Naidu:** Conceptualization, Resources, Writing – review & editing; **Halimah Sa'diyah:** Investigation, Data curation; **Rizky Novia Saputri:** Investigation, Resources; **Jagad Aditya Dewantara:** Investigation, Resources; **Patmisari:** Investigation, Data curation.

## Declaration of Competing Interest

The authors declare that they have no known competing financial interests or personal relationships that could have appeared to influence the work reported in this paper.

## Data Availability

Dataset for Measuring Adversity Quotient, Digital Competence, Attitude Computer Technology and Self-Efficacy (Reference data) (Mendeley Data).
